# scHiGex: predicting single-cell gene expression based on single-cell Hi-C data

**DOI:** 10.1093/nargab/lqaf002

**Published:** 2025-01-27

**Authors:** Bishal Shrestha, Andrew Jordan Siciliano, Hao Zhu, Tong Liu, Zheng Wang

**Affiliations:** Department of Computer Science, University of Miami, Coral Gables, FL 33146, United States; Department of Computer Science, University of Miami, Coral Gables, FL 33146, United States; Department of Computer Science, Florida Memorial University, Miami Gardens, FL 33504, United States; Department of Computer Science, University of Miami, Coral Gables, FL 33146, United States; Department of Computer Science, University of Miami, Coral Gables, FL 33146, United States

## Abstract

A novel biochemistry experiment named HiRES has been developed to capture both the chromosomal conformations and gene expression levels of individual single cells simultaneously. Nevertheless, when compared to the extensive volume of single-cell Hi-C data generated from individual cells, the number of datasets produced from this experiment remains limited in the scientific community. Hence, there is a requirement for a computational tool that can forecast the levels of gene expression in individual cells using single-cell Hi-C data from the same cells. We trained a graph transformer called scHiGex that accurately and effectively predicts gene expression levels based on single-cell Hi-C data. We conducted a benchmark of scHiGex that demonstrated notable performance on the predictions with an average absolute error of 0.07. Furthermore, the predicted levels of gene expression led to precise categorizations (adjusted Rand index score 1) of cells into distinct cell types, demonstrating that our model effectively captured the heterogeneity between individual cell types. scHiGex is freely available at https://github.com/zwang-bioinformatics/scHiGex.

## Introduction

Gene expression is a key intermediate level in the intricate process of transcription and translation of genetic code into discernible phenotypes, influencing cellular functions and acquiring a wide range of cell identities [1]. With sophisticated control mechanisms involving transcription factors and epigenetic modifications, cells govern the temporal, spatial, and quantitative aspects of gene expression [2, 3]. This precision ensures proper biological function and adaptive responses to environmental stimuli, maintaining cellular homeostasis [4]. Dysregulation of gene expression has been indicated in various diseases, including cancers, genetic disorders, and neurodegenerative conditions—making comprehension of gene expression crucial for developing therapeutic interventions [5].

The chromosome conformation capture (3C) method, pioneered to elucidate spatial proximities between individual genomic loci within a cell population, has significantly evolved through subsequent advancements [6]. The core process of 3C technologies involves cross-linking chromatin, digestion with a restriction enzyme, and subsequent ligation of spatially proximate DNA fragments. The 3C-on-chip technique emerged to extend the scope, allowing the capture of spatial proximities between a specific locus and all other genomic loci. Further, the 3C-carbon copy was developed to explore interactions between all restricted fragments within a defined genomic region, followed by the Hi-C experiment [7] that revolutionized the field by comprehensibly capturing spatial proximities across the entire genome within a cell population.

Hi-C’s ability to capture the spatial relationships between genomic elements, including the identification of topologically associated domains as pivotal structural and functional units of the genome [8] and other higher-order chromatin structures such as chromatin loops and compartments, provides a comprehensive understanding of the regulatory landscape of the genome [9]. This breakthrough also enables the computational reconstruction of chromosomes’ three-dimensional (3D) architectures [10]. This information is instrumental in unraveling the complex mechanisms that govern gene expression, offering a holistic view of how the genome is organized and orchestrated to regulate cellular processes [11].

In contrast to the conventional or bulk Hi-C technique, which captures genome-wide chromatin interactions over a population of cells, single-cell Hi-C allows the analysis of chromatin interactions at the resolution of individual cells, providing a more nuanced understanding of the genome with insight into heterogeneity between cells and uncovering cell-to-cell variability in chromatin conformation [12]. Analogous to single-cell Hi-C, single-cell RNA sequencing (scRNA-seq) reveals that gene expression is heterogeneous in similar cell types [13].

Recent advances in single-cell multi-omics sequencing technologies have elevated our understanding of cells at the molecular level by enabling parallel profiling between various omics within a single cell: transcriptomics, genomics, epigenomics, proteomics, and metabolomics [14]. A new molecular assay, HiRES, has enabled the simultaneous capture of single-cell chromatin conformation and scRNA-seq in RNA-centric multi-omics technology, filling a long-standing research void [15]. This assay avoids the physical separation of genome and transcriptome, in contrast to conventional methods that utilize distinct DNA and RNA isolation protocols, allowing combined analysis of chromatin interaction and gene expression within the same cell with more comprehensive and reliable data.

To the best of our knowledge, a computational tool has not been available so far for predicting single-cell gene expression using simultaneously captured single-cell Hi-C data. While our approach focuses on gene expression prediction using single-cell Hi-C, related works, such as the study by Zhang *et al.* on gene co-expression and bulk Hi-C data, highlights the novelty of our method [16]. To fill this gap, we successfully used the state-of-the-art graph transformer in deep learning to develop a predictive model demonstrating notable performance. The evaluation results highlight the accuracy and effectiveness of our tool, showcasing substantial advancements in single-cell gene expression prediction using single-cell chromatin conformation interaction.

## Materials and methods

### Overview

**Figure 1.**
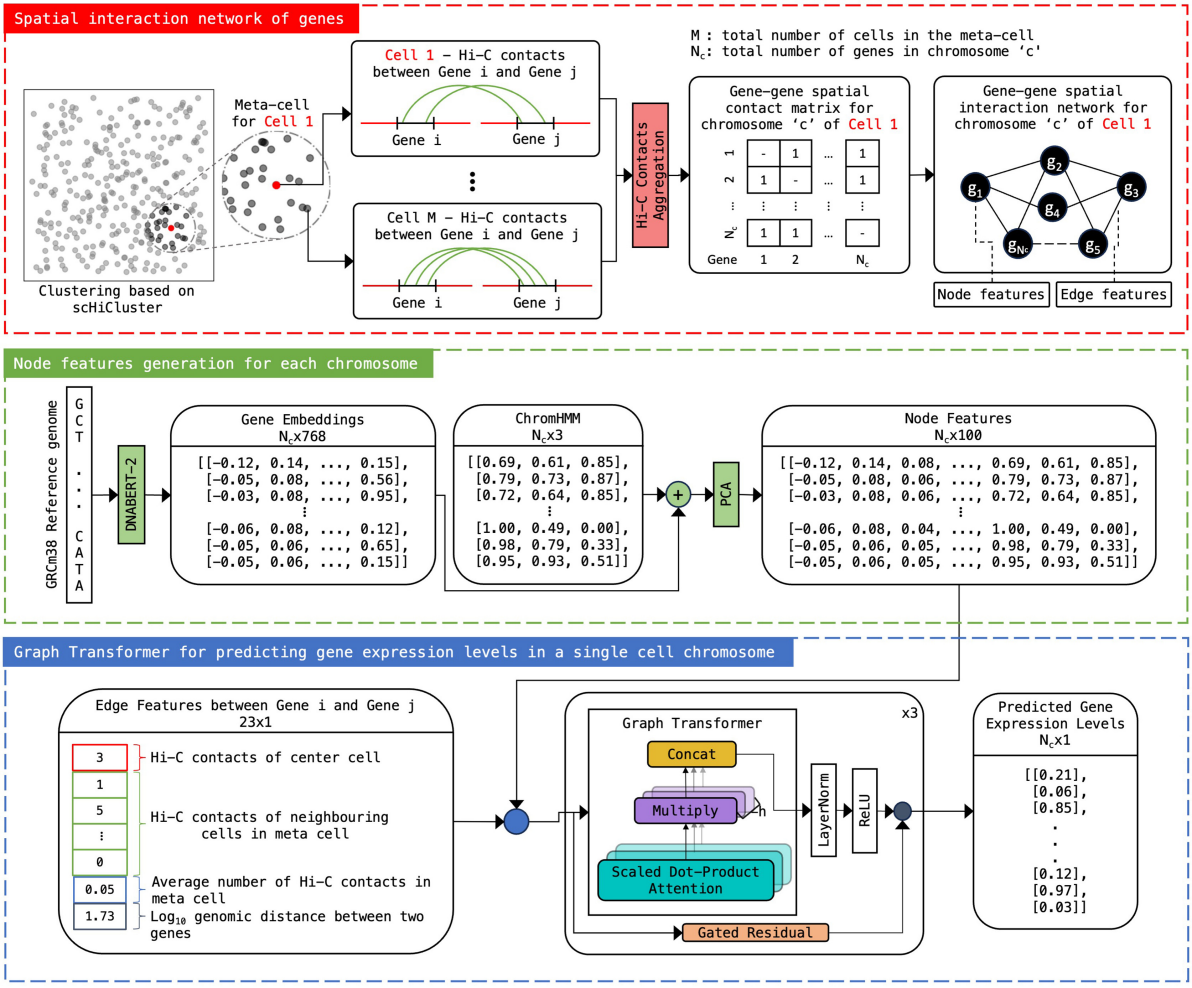
Architectural overview of scHiGex, illustrating the process of node feature generation through data manipulation, Hi-C processing for spatial interaction network creation, and the graph transformer architecture employed for predicting gene expression.

The architecture of scHiGex in Fig. 1 illustrates the methodologies used for gene expression prediction using the graph transformer [17]. The GRCm38 reference genome was utilized to create DNA sequence embeddings using DNABERT-2 [18]. The DNA sequence embedding in combination with the full-stack chromHMM [19] was subjected to principal component analysis (PCA) to generate node features for the genes. The single-cell Hi-C data were utilized to create Hi-C contact matrices showing contacts between genes in their respective cell. Using these Hi-C contact matrices, a gene–gene spatial network was constructed indicating the spatial interaction between different genes. Later sections will provide a thorough explanation of the steps involved in generating the node features and edge features that were then fed into the graph transformer network.

### Datasets

In this research, simultaneously captured single-cell Hi-C data and gene expressions from the same cells captured by the HiRES protocol (GSE223917) [15] were used. The simultaneous single-cell Hi-C and scRNA-seq profiling were done on developing mouse embryo cells and brain cells with reads mapped to the GRCm38 reference genome. These two datasets comprised 21 distinct cell types from the embryo and 7 distinct cell types from the brain, from which we selected 10 cell types and 6 cell types with the highest number of cells, respectively. The cells of disjoint cell types were used for training, validation, and blind tests to examine the generalizability and differentiating capacity of the trained model on different cell types. The genes with at least one gene expression value were only considered in this experiment to reduce redundancy. In the experiment, each graph is a data instance that was built on a chromosome of an individual cell, where nodes represent genes and the edges are defined by the Hi-C contacts between the genes.

For the embryo dataset, the training set was composed of 66 696 graphs of the cell types: blood, mitosis, early neurons, mix late mesenchyme, and ExE endoderm. The validation set included 18 606 graphs, consisting of the following cell types: radial glia and early mesoderm. The blind test set was composed of 27 048 graphs, which contained the following cell types: neural ectoderm, early mesenchyme, and ExE ectoderm.

Similarly, for the brain dataset, the training set was composed of 5734 graphs, consisting of the following cell types: Ex1, In1, and Oli. The validation set included 903 graphs, consisting of Ex2 cells, and the blind test set was composed of 1407 graphs, which contained the Ast and In2 cell types. The brain dataset was considered in this experiment despite the number of graphs in the brain dataset being significantly less than the embryo dataset to assess the model’s performance on limited data and another biological context.

The gene expression values ranged from 0 to over 1000, with a higher density of values near 0. To minimize the effect of outliers and ensure the focus on relevant variations, the gene expression values were clipped between 0 and 3 (98th percentile) and then were subjected to min–max normalization.

### Building meta-cells for single-cell Hi-C data

The single-cell Hi-C data are sparse, hence making the gene–gene spatial interaction network dilute. To overcome this sparsity, the concept of meta-cell [20] was used as the data imputation technique. To construct the meta-cell for each target cell, the top 20 neighboring cells with the most similar Hi-C pattern are grouped along with the target cell as implemented in [20]. Since the clustering is done based on the similarity of the Hi-C pattern, the population of cells taken into consideration for the construction of meta-cells is of the same cell type as of target cell type. We also developed another version without meta-cells for comparison. Further information is provided in the Supplementary data.

### Gene–gene spatial interaction network

In the gene–gene spatial interaction network, each gene is represented as a node. To define edges in the gene–gene spatial interaction networks, Hi-C contacts for each chromosome of the meta-cell are aggregated. An edge was created between two genes if the two gene regions had at least one Hi-C contact in the meta-cell. Due to the limited number of interchromosomal Hi-C contacts, only intrachromosomal Hi-C contacts were considered to build the gene–gene spatial interaction network. The gene regions were defined using the GENCODE release M23 (based on GRCm38) gene annotation [21].

### Node and edge features

The DNA nucleotide sequence of the GRCm38 genome assembly was used to generate the embeddings for each gene using DNABERT-2 [18], effectively capturing the semantic and structural patterns within genomic sequences. Specifically, GENCODE release M23 [21] was used to extract the nucleotide sequence for each gene. The nucleotide sequence for each gene was segmented into 1000 base pairs and passed as input into DNABERT-2 with mean pooling, which outputted a set of embeddings of 768 real numbers for each gene. The multiple embeddings from the segments of the same gene were averaged along the dimension 0 giving the final gene embedding of size 768 for each gene.

In addition to this, the full-stack chromHMM genome annotation was used to extract the chromatin state information for each gene [19]. The embeddings from the DNABERT-2 and chromHMM were concatenated together, resulting in a 771-dimensional feature space. To enhance computational efficiency and reduce the risk of overfitting due to the high dimensionality, PCA was performed to reduce the dimensionality to 100 components, capturing 91.77% of the total variance. The resulting lower-dimensional embeddings formed the node features of genes for the experiment.

The edge features between two genes were generated using the Hi-C contact matrices in the meta-cell and their one-dimensional (1D) genomic distance. Initially, the first edge features consist of 21 integers that represent the Hi-C contacts between the two genes in each cell in the meta-cell. Then, the average of the Hi-C contacts was calculated and used as the next feature, followed by their log_10_ genomic distance in the 1D nucleotide sequence calculated from their midpoint.

### Graph transformer

The input node features for the *o*th block of the graph transformer are represented as $G^{(o)}={g_1^{(o)},\ g_2^{(o)},\ \ldots ,\ g_n^{(o)}}$, where *g* denotes gene and *n* is the total number of genes in the chromosome of study. As defined in [17], for the *h*th head attention of each edge from *i* to *j*, the query vector $q_{h,i}^{(o)}$, the key vector $k_{h,j}^{(o)}$, and the value vector $v_{h,j}^{(o)}$ are generated as


\begin{eqnarray*} q_{h,i}^{(o)} = W_{h,q}^{(o)}g_i^{(o)} + b_{h,q}^{(o)} ,\end{eqnarray*}

\begin{eqnarray*} k_{h,j}^{(o)} = W_{h,k}^{(o)}g_j^{(o)} + b_{h,k}^{(o)}, \end{eqnarray*}

\begin{eqnarray*} v_{h,j}^{(o)} = W_{h,v}^{(o)}g_j^{(o)} + b_{h,v}^{(o)} ,\end{eqnarray*}

where $W_{h,q}^{(o)}$, $W_{h,k}^{(o)}$, $W_{h,v}^{(o)}$, $b_{h,q}^{(o)}$, $b_{h,k}^{(o)}$, and $b_{h,v}^{(o)}$ are trainable parameters.

Similarly, the edge feature is encoded as


\begin{eqnarray*} e_{h,ij}^{(o)} = W_{h,e}^{(o)}e_{ij}^{(o)} + b_{h,e}^{(o)} ,\end{eqnarray*}

in which $W_{h,e}^{(o)}$ and $b_{h,e}^{(o)}$ are trainable parameters as well.

Different from [17], we also computed the attention score using only the encoded edge and performed a gated attention head. This has been done to ensure that our model encompasses edge knowledge into multi-head attention for each block. The multi-head attention is calculated as follows:


\begin{eqnarray*} \alpha 1_{h,ij}^{(o)}= \frac{\bigl \langle q_{h,i}^{(o)}, e_{h,ij}^{(o)} \bigr \rangle }{\sum _{u\in N(i)} \bigl \langle q_{h,i}^{(o)}, e_{h,iu}^{(o)} \bigr \rangle }, \end{eqnarray*}

\begin{eqnarray*} \alpha 2_{h,ij}^{(o)}= \frac{\bigl \langle q_{h,i}^{(o)}, k_{h,j}^{(o)} + e_{h,ij}^{(o)} \bigl \rangle }{\sum _{u\in N(i)} \bigl \langle q_{h,i}^{(o)}, k_{h,u}^{(o)} + e_{h,iu}^{(o)} \bigr \rangle }, \end{eqnarray*}

\begin{eqnarray*} \rho _{h,ij}^{(o)} = \mathrm{sigmoid}(W_{l}^{(o)} [ \alpha 1_{h,ij}^{(o)};\ \alpha 2_{h,ij}^{(o)};\ \alpha 1_{h,ij}^{(o)} - \alpha 2_{h,ij}^{(o)}] ) ,\end{eqnarray*}

\begin{eqnarray*} \alpha _{h,ij}^{(o)} = \rho _{h,ij}^{(o)} \alpha 1_{h,ij}^{(o)} + (1-\rho _{h,ij}^{(o)}) \alpha 2_{h,ij}^{(o)}, \end{eqnarray*}

in which $\bigl \langle q,e \bigr \rangle$ and $\bigl \langle q,k+e \bigr \rangle$ are the exponential dot-product functions and calculated as $\bigl \langle q,e \bigr \rangle = {\rm e}^{{q^Te}/{\sqrt{d}}}$ and $\bigl \langle q,k+e \bigr \rangle = {\rm e}^{{q^T(k+e)}/{\sqrt{d}}}$, respectively. The *d* represents each attention head’s hidden size.

After calculating the multi-head attention and the value vector, the message aggregation from the neighborhood node *j* to source node *i* is performed as


\begin{eqnarray*} \widehat{g}_i^{(o+1)} = \Vert _{h=1}^H \left[\sum _{j \in N(i)} \alpha _{h,ij}^{(o)}(v_{h,j}^{(o)} + e_{h,ij})\right] ,\end{eqnarray*}

where $\Vert _{h=1}^H$ represents the concatenation operation on all the head attention.

As performed in [17], a gated residual connection between layers is performed to prevent over-smoothing.


\begin{eqnarray*} r_i^{(o)} = W_r^{(o)}g_i^{(o)}+b_r^{(o)} ,\end{eqnarray*}

\begin{eqnarray*} \beta _i^{(o)} = \mathrm{sigmoid}(W_g^{(o)}[\widehat{g}_i^{(o+1)};\ r_i^{(o)};\ \widehat{g}_i^{(o+1)}-r_i^{(o)}]) ,\end{eqnarray*}

\begin{eqnarray*} g_i^{(o+1)} = \mathrm{ReLU}(\mathrm{LayerNorm}( (1-\beta _i^{(o)})\widehat{g}_i^{(o+1)} + \beta _i^{(o)}r_i^{(o)})). \end{eqnarray*}

As implemented in [20], we aggregated the multi-head attention with the edge features:


\begin{eqnarray*} \widehat{e}_{ij}^{(o+1)} = \Vert _{h=1}^H(\alpha _{h,ij}^{(o)}, e_{ij}^{(o)}). \end{eqnarray*}

As employed in [17, 20], for the last output layer, averaging for multi-head output is done instead of concatenation as below:


\begin{eqnarray*} \widehat{g}_i^{(o+1)} = \frac{1}{H}\sum _{h=1}^{H}\left[\sum _{j \in N(i)} \alpha _{h,ij}^{(o)}(v_{h,j}^{(o)} + e_{h,ij}^{(o)})\right] .\end{eqnarray*}

The detailed architecture of the graph transformer and a complete summary of the notations used in this section are presented in Supplementary Fig. S1 and Supplementary Table S1, respectively.

### Implementation details

The graph transformer was trained on the modified version of the TransformerConv [17] implemented on PyTorch [22]. A hyperparameter search was conducted on key parameters such as learning rate, batch size, and number of heads. Our model performed best with a learning rate of 0.0001, three transformer blocks, eight instances per batch, and the number of heads set to 10. Details on hyperparameter sensitivity analysis can be found in Supplementary Table S2. Model parameters were optimized using AdamW [23], with early stopping based on validation loss and patience of 5. A ReduceLROnPlateau scheduler, with a reduction factor of 0.5 and patience of five epochs, was employed to adjust the learning rate. The models were trained on a 40 GB NVIDIA A100 GPU with a training speed-up of 180 times compared to training on a CPU, which would take ∼48 h per epoch, whereas the GPU reduced this to ∼16 min per epoch. Similarly, the inference time was reduced from 4.5 s per graph on a CPU to 0.017 s per graph on a GPU.

### Loss function

The distribution of gene expression data is highly skewed toward zero, making the distribution unbalanced: ∼92% of observations contain zero values. This imbalance may lead to biases in the model with an inclination toward zero values, potentially overshadowing the importance of nonzero values.

To overcome this challenge, we implemented two strategies when computing the loss during the training phase: downsampling and weighted L1 loss computation. The downsampling of the zero targets was done to match with the number of nonzero values to achieve a balanced distribution. In addition, using the weighted L1 loss, different weights were assigned to zero and nonzero values. Through experimentation, a weighted ratio of 4:6 for zero to nonzero values was determined to give optimal performance in combination with downsampling.

Additionally, for the model to learn the true gene expression distribution (a high peak at the zero value with a few other smaller peaks at other values, which will be shown in the “Results” section), the standard L1 loss without downsampling was used but only with the probability of .1. Specifically, each batch during the training process contains eight graphs, and for every batch of training examples, we generated a random number between 0 and 1. If the random number was <0.1, we used the standard L1 loss on the unbalanced version of the batch of training examples (no downsampling performed) as below:


\begin{eqnarray*} L = \frac{1}{N}\sum _{i=1}^{N}|y_\mathrm{true}-y_\mathrm{predicted}| .\end{eqnarray*}

If the random number was ≥0.1, we used a weighted L1 loss on a balanced batch of training example as below:


\begin{eqnarray*} L_\mathrm{zero} = 0.4 \times \sum _{i=1}^{\widehat{N}}|\widehat{y}_\mathrm{true}-\widehat{y}_\mathrm{predicted}|,\quad \forall \ \widehat{y}_\mathrm{true}=0 ,\end{eqnarray*}

\begin{eqnarray*} L_\mathrm{nonzero} = 0.6*\sum _{i=1}^{\widehat{N}}|\widehat{y}_{{\rm true}}-\widehat{y}_\mathrm{predicted}|,\quad \forall \ \widehat{y}_\mathrm{true}>0 ,\end{eqnarray*}

\begin{eqnarray*} L = \frac{1}{\widehat{N}}(L_\mathrm{zero} + L_\mathrm{nonzero}) ,\end{eqnarray*}

in which *y*_true_ and *y*_predicted_ are the true and predicted gene expression values, respectively; $\widehat{y}_\mathrm{true}$ and $\widehat{y}_\mathrm{predicted}$ are the true and predicted gene expression values after downsampling, respectively; *N* is the total number of observations; and $\widehat{N}$ is the number of observations after downsampling.

This combined approach ensured that the model accurately learned the true distribution meanwhile not exhibiting biases toward zero, resulting in better performance than if only the standard L1 loss or the weighted L1 loss on a balanced dataset was used (results not shown).

During the validation and blind test phase, the standard L1 loss without downsampling was computed to make the evaluation of the model’s performance deterministic.

### Evaluation

#### Naive predictor

As there are no other published tools to compare the performance with, we constructed two naive predictors. Both rely solely on the gene expressions of the neighboring nodes in the gene–gene spatial interaction networks to make inference. Naive Predictor 1 predicts the gene expression by calculating the average gene expression levels of the genes that are one hop away in the graph. Naive Predictor 2 predicts the gene expression by calculating the weighted average of the gene expression levels of the genes that are one hop away in the graph, where the weights are the log_10_ genomic distance between the genes.

#### Performance metrics

The performance of scHiGex was evaluated by measuring various metrics between the predictions and ground truths: the accuracy, average precision (AP), *F*1 score, Pearson’s correlation (PCC), Matthew’s correlation (MCC), Spearman’s correlation (SCC), the area under the receiver operating characteristic (ROC) curve (AUC), and average L1 loss. The accuracy was calculated as the sum of observations with absolute difference between prediction and ground truth <0.1 divided by the total number of observations.

#### Cell type classification and its evaluation

The *K*-means clustering technique was applied to the target and predicted gene expression levels of all genes of the cells. The number of clusters was selected to match the total number of unique cell types in the input. To quantify the similarities between the true cell types and the clustering results, we calculated the adjusted Rand index (ARI) as an evaluation metric, which was referred as ARI-global. An ARI score near zero implies that the categorization is arbitrary, whereas an ARI value of 1 shows that the clustering is completely pure.

To visualize the clusters of cells from the target and predicted gene expression levels for all genes, we utilized the Uniform Manifold Approximation and Projection for Dimension Reduction (UMAP) algorithm [24]. This algorithm generated two features, which were used as the axes to plot the cells in a two-dimensional Cartesian coordinate system. Similar to ARI-global, using the two-dimensionally reduced features from UMAP, the ARI-reduced score was also computed.

## Results

The evaluation results on both embryo and brain blind test cells based on eight evaluation metrics are presented in Table 1. The blind test is conducted on four different models for both embryo and brain datasets: scHiGex, scHiGex_ind, and the naive predictors. The evaluation was done on 10 repeated runs to check the consistency of the model’s learning ability. Detailed results are provided in Supplementary Tables S3–S6. Here, scHiGex_ind is the model that does not have meta-cell applied while constructing the gene–gene spatial interaction network. scHiGex_ind is evaluated to validate the contribution of meta-cell in the model.

**Table 1.** The evaluation results for scHiGex, scHiGex_ind, and the naive predictor on the blind test for both embryo and brain cells

Sample	Model	Accuracy	AP	*F*1 score	PCC	MCC	SCC	AUC	Avg. abs. error
Embryo	scHiGex	0.80 ± 0.00	0.54 ± 0.01	0.49 ± 0.01	0.59 ± 0.01	0.46 ± 0.01	0.40 ± 0.00	0.89 ± 0.00	0.07 ± 0.00
	scHiGex_ind	0.76 ± 0.01	0.48 ± 0.01	0.43 ± 0.01	0.53 ± 0.01	0.41 ± 0.01	0.39 ± 0.00	0.88 ± 0.00	0.08 ± 0.00
	Naive Predictor 1	0.29	0.14	0.26	0.04	0.17	0.13	0.61	0.22
	Naive Predictor 2	0.45	0.15	0.28	0.03	0.19	0.16	0.64	0.19
Brain	scHiGex	0.76 ± 0.01	0.55 ± 0.02	0.53 ± 0.01	0.57 ± 0.03	0.44 ± 0.01	0.44 ± 0.01	0.85 ± 0.01	0.09 ± 0.00
	scHiGex_ind	0.69 ± 0.01	0.51 ± 0.01	0.48 ± 0.01	0.52 ± 0.01	0.39 ± 0.00	0.43 ± 0.00	0.84 ± 0.00	0.12 ± 0.01
	Naive Predictor 1	0.23	0.20	0.32	0.09	0.15	0.17	0.62	0.27
	Naive Predictor 2	0.36	0.21	0.34	0.06	0.19	0.18	0.64	0.24

scHiGex and scHiGex_ind underwent testing through 10 repeated training runs, with the standard error being reported. The naive predictor (baseline) does not have a standard error as it does not have training stage. scHiGex_ind is referred to as the model that does not consider meta-cell while constructing the gene–gene spatial interaction network.

The results show that scHiGex outperforms both scHiGex_ind and the naive predictors (baseline models) in all evaluation metrics for both datasets. The performance of both scHiGex and scHiGex_ind is consistent across the repeated runs, with the standard error being <0.01. This demonstrates the ability of the graph transformer to capture data patterns and graph topology in the networks in a consistent manner. Moreover, scHiGex is able to perform better than the scHiGex_ind model, indicating the importance of meta-cell in constructing the gene–gene spatial interaction network.

In addition to these models, various other modifications were explored on the scHiGex_ind version, including alterations to the loss function and increases in flank size. However, these changes did not result in any notable improvements in model performance (results not provided).

scHiGex demonstrates significantly improved performance over the naive predictors. The average per-graph Wilcoxon signed-rank test *P*-values were calculated between the absolute errors of scHiGex and the naive predictors. For the embryo dataset, the *P*-values were .017 and .018 for Naive Predictor 1 and Naive Predictor 2, respectively. For the brain dataset, the *P*-values were .013 and .016 for Naive Predictor 1 and Naive Predictor 2, respectively. These results indicate a statistically significant difference, highlighting that scHiGex consistently outperforms both naive models across different datasets.

The absolute difference between the ground truth and predicted gene expression values from scHiGex exhibited overall standard deviations of 0.16 and 0.19 on the embryo and brain datasets, respectively, which were notably lower than those of Naive Predictor 1 (0.21 and 0.23) and Naive Predictor 2 (0.23 and 0.25) on the same datasets, with *P*-values <.0001. This reduction in variability underscores the enhanced consistency of scHiGex’s predictions compared to both naive models. As illustrated in Supplementary Fig. S2, the violin plots depict this reduced variability and also reveal that the error distribution for scHiGex is more tightly concentrated around zero, indicating greater predictive accuracy.

Confidence interval analysis of the absolute differences between the ground truth and predicted gene expression values further supports these findings. The confidence intervals for scHiGex are both non-overlapping and lower than those of the naive predictors, indicating that scHiGex’s predictions are consistently closer to the actual gene expression levels. The results of the confidence interval analysis are provided in Supplementary Table S7.

**Figure 2.**
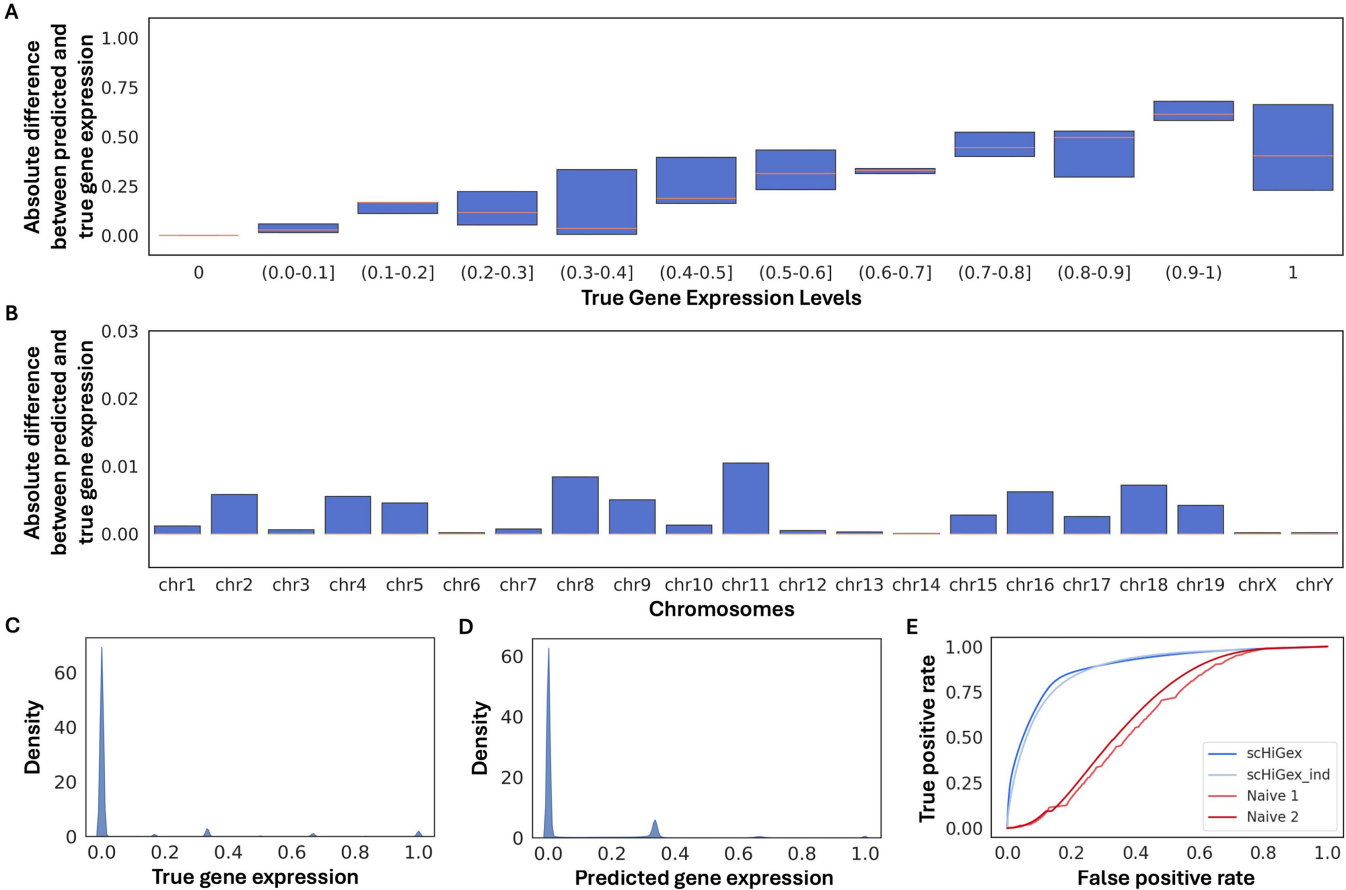
Evaluation plots of blind test on embryo cells using scHiGex. (**A**, **B**) Box plots of absolute difference between the predicted and true gene expression levels on the blind test cells and each chromosome, respectively. (**C**, **D**) Distribution of the true and predicted gene expression levels on the blind test cells. (**E**) ROC curve of scHiGex as compared to scHiGex_ind and the naive predictors on the blind test cells.

To conduct a more detailed analysis of our model’s performance (scHiGex) on the embryo dataset, we plotted the absolute difference between the predicted and actual gene expression levels on the blind test cells (see Fig. 2A). It is evident that scHiGex can predict gene expression values with a small absolute discrepancy, with a median close to 0, demonstrating reliable capacity to determine gene expression levels close to the actual values.

Figure 2B displays the absolute difference between the predicted and actual gene expression levels for each chromosome. The absolute difference is uniformly distributed across all chromosomes, with the median absolute differences near zero.

The distribution plot in Fig. 2C clearly shows a higher concentration of zero gene expression values, as supported by [25], where only 10% of the gene expression values in the blind set are nonzeros. Figure 2D demonstrates that the predicted gene expression level distribution closely resembles the actual gene expression level distribution.

Figure 2E displays the ROC curves made by scHiGex, scHiGex_ind, and the naive predictors on the blind test cells with the AUC values of 0.89, 0.88, 0.61, and 0.64, respectively.

**Figure 3.**
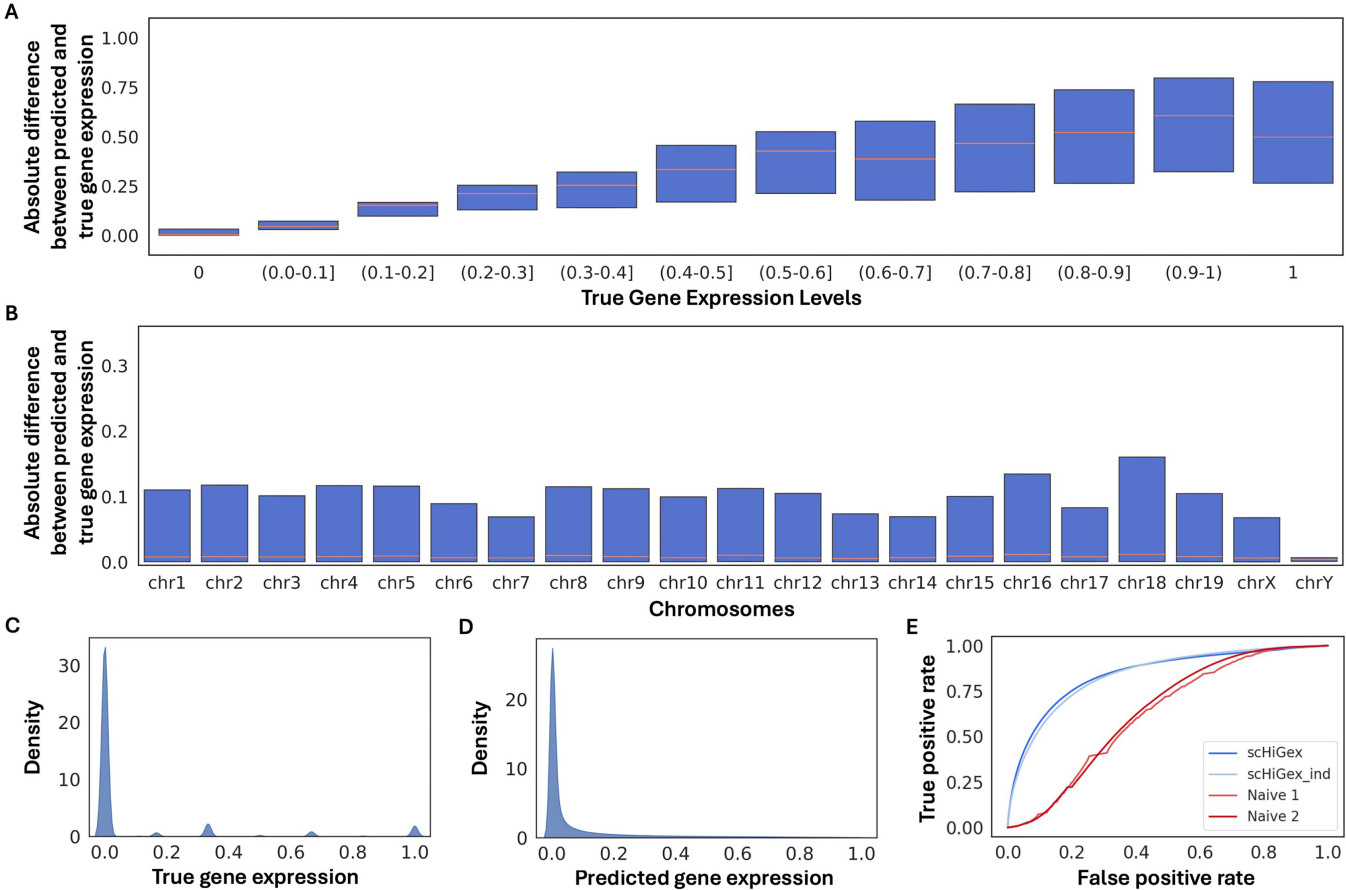
Evaluation plots of blind test on brain cells using scHiGex. (**A**, **B**) Box plots of absolute difference between the predicted and true gene expression levels on the blind test cells and each chromosome, respectively. (**C**, **D**) Distribution of the true and predicted gene expression levels on the blind test cells. (**E**) ROC curve of scHiGex as compared to scHiGex_ind and the naive predictors on the blind test cells.

Similarly, Fig. 3 illustrates the blind test evaluation plots for scHiGex when tested on the brain dataset. Despite outperforming scHiGex_ind and the naive predictor, Fig. 3A and B shows that the absolute difference between the predicted and actual gene expression levels is slightly higher than that in the embryo dataset, indicating the effect of limited data while training the model. A similar pattern in the distribution of gene expression levels can be seen in Fig. 3C and D. The ROC curve and AUC values of 0.85, 0.84, 0.62, and 0.64 for the predictions made by scHiGex, scHiGex_ind, and the naive predictors, respectively, on the blind test cells are displayed in Fig. 3E.

The blind test performance evaluation plot of both embryo and brain cells using naive predictors is provided in Supplementary Figs S3–S6. These results show that the naive predictors perform poorly compared to scHiGex and scHiGex_ind, indicating the effectiveness of our model in predicting gene expression levels using single-cell Hi-C data.

**Figure 4.**
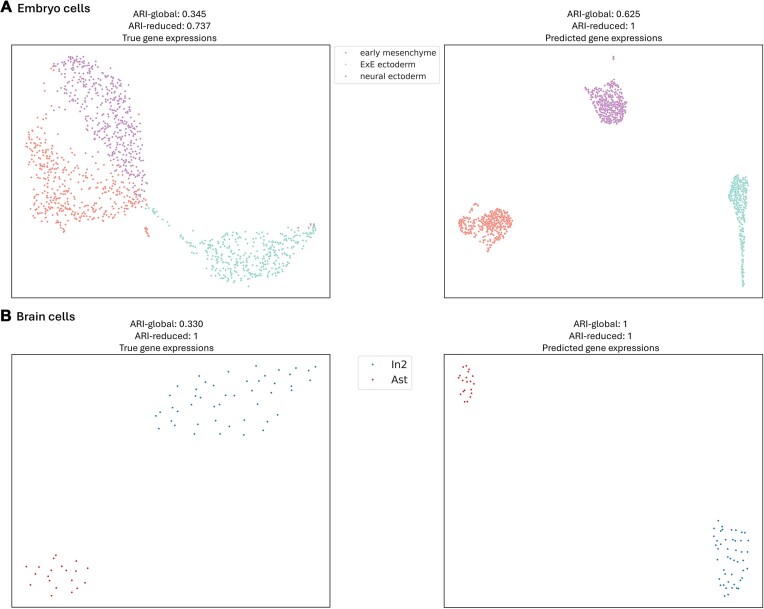
Visualization of dimensionally reduced two feature components generated using UMAP for the clustering results based on the true (left) and predicted (right) gene expression levels of the blind test cells: (**A**) embryo cells and (**B**) brain cells. The ARI-global and ARI-reduced scores are reported for both datasets.

Moreover, the visualization of the blind test cells based on the true and predicted gene expression levels using UMAP is shown in Fig. 4. The results demonstrate that scHiGex can correctly categorize cells into distinct cell types based on the predicted gene expression levels, as evidenced by the clear separation of the cells into different clusters with an ARI-reduced score of 1, indicating that the clustering results are completely pure. This suggests that our model effectively captures the heterogeneity between individual cell types.

Interestingly, since the ARI-global and ARI-reduced scores from predicted gene expression levels are greater than the ARI-global and ARI-reduced scores from true gene expression levels, it suggests that the model has learned meaningful features from the gene expression levels to categorize the cells into distinct cell types while filtering out the noise.

## Discussion and conclusions

In this paper, we present scHiGex, a computational tool that predicts single-cell gene expression levels using single-cell Hi-C data. By utilizing the cutting-edge deep learning algorithm graph transformer, our method exhibited significant performance across several evaluation parameters.

scHiGex incorporates two categories of data in a graphical format: node features, which represent genes using DNA sequence data, and edge features, which encapsulate spatial organization between genes using single-cell Hi-C data. All the cells, regardless of their type, have identical DNA sequence inputs, which are obtained from the same reference genome. Therefore, relying just on DNA sequence data would result in consistent predictions for all cell types, making cell type categorization pointless. Nevertheless, our approach attains perfect categorization of cell types, highlighting the accuracy and effectiveness of our trained graph transformer in identifying complex patterns within data organized in a graph structure. Furthermore, this supports the notion that our forecasted gene expressions maintain the natural differences between cells and the unique traits linked to each cell type, as observed in single-cell Hi-C data [12].

The results from scHiGex indicate a substantial relationship between the 3D structure of individual cells and the expression of genes. However, although our data suggest the existence of a connection between them, further research is necessary to clarify the exact processes and effects that exist between them. Despite the strong predictive performance of scHiGex, the graph transformer model functions as a “black box,” making it challenging to decipher how specific genomic features influence predictions [26, 27]. Therefore, using interpretable artificial intelligence methods could provide insights into feature importance and help validate key interactions experimentally, bridging the gap between predictive accuracy and biological understanding.

## Supplementary Material

lqaf002_Supplemental_File

## Data Availability

The source code of scHiGex is freely available on GitHub at https://github.com/zwang-bioinformatics/scHiGex/ and on Zenodo at https://doi.org/10.5281/zenodo.14613245.
