# Supplementary material for: scHiGex: predicting single-cell gene expression based on single-cell Hi-C data
Source: NAR Genom Bioinform. 2025 Jan 27;7(1):lqaf002. doi: 10.1093/nargab/lqaf002 (PMC11770341; doi:10.1093/nargab/lqaf002)
Supplement: lqaf002_Supplemental_File [file lqaf002_supplemental_file.pdf]

Supplementary document for

scHiGex: predicting single-cell gene expression based on single-cell Hi-C data

Bishal Shrestha, Andrew Jordan Siciliano, Hao Zhu, Tong Liu and Zheng Wang*

Department of Computer Science, University of Miami, Coral Gables, 33143, Florida, USA

*To whom correspondence should be addressed.

1 Methods

1.1 Building meta-cells for single-cell Hi-C data

The intrachromosomal contact matrix for each chromosome was generated using single-cell Hi-C at a 1 Mb resolution. We utilized a tool named scHiCluster [1] on the generated intrachromosomal Hi-C contact pairs across all cells. For each chromosome, the intrachromosomal contact matrix was processed with scHiCluster to obtain an output of n x $(n-1)$ components, where n represents the number of cells and n-1 represents the number of components per cell.

Principal component analysis (PCA) was performed using the sklearn package on the flattened gene-gene contact matrix of each chromosome to extract the top 40 principal components. These 40 principal components from all chromosomes were concatenated along axis=1, resulting in an n x 40*n_chr matrix, where n_chr denotes the number of chromosomes. PCA was then reapplied with n_components set to min(n, 40*n_chr) – 1.

The output was subsequently used to compute the Euclidean distance between all cell pairs within the same cell type. We identified the 20 nearest neighboring cells within the same cluster as the target cell, making a meta cell for the target cell. These selected cells were then combined to aggregate the Hi-C contacts for the target cell, resulting in the aggregated Hi-C contact matrix where the edges were created if at least one cell from the meta-cell contained an edge. The contacts from all the cells in the meta-cell were used to generate the edge features (explained in the manuscript).

1.2 Graph Transformer Architecture

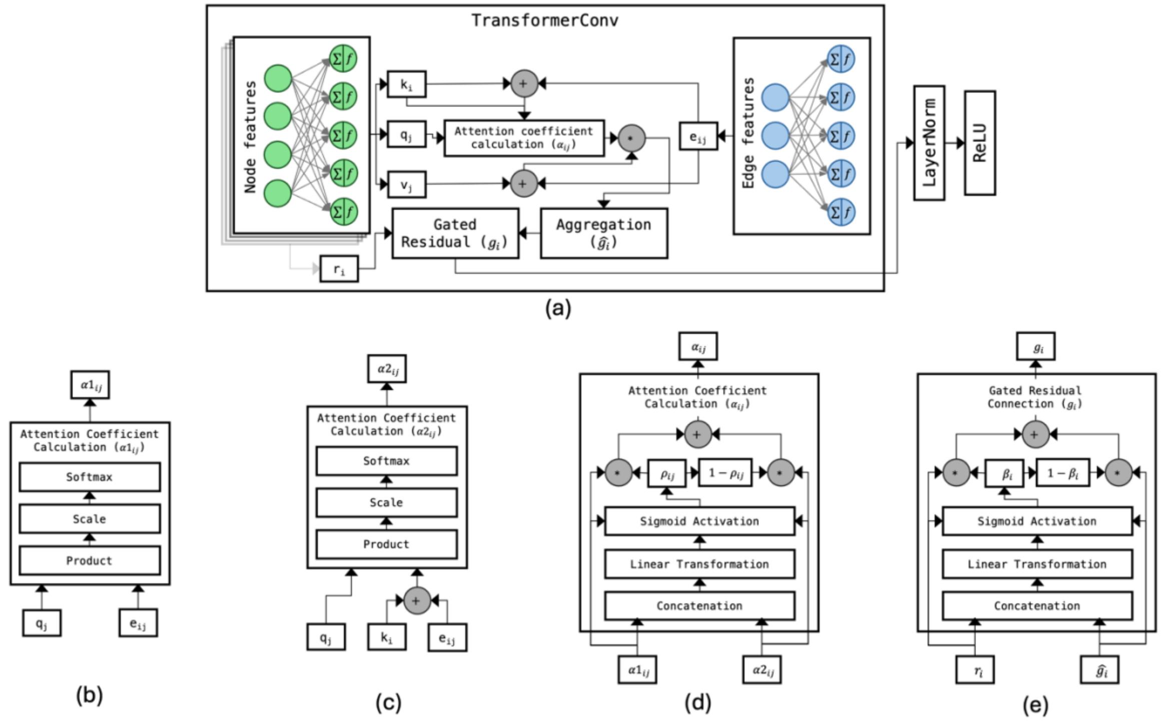

Figure S1: Overview of the Graph Transformer architecture implemented in the scHiGex for the o-th block. (a) General architecture with attention coefficient calculations, gated residuals, and aggregation of features. (b) Attention coefficient calculation ($\alpha 1_{ij}$) using query and edge features. (c) Combined node-edge attention coefficient ($\alpha 2_{ij}$) calculation, integrating query, key, and edge features. (d) Final attention coefficient (α_{ij}), combining $\alpha 1_{ij}$ and $\alpha 2_{ij}$ through a gated mechanism. (e) Gated residual connection (g_i), using the initial node representation (r_i) with the aggregated representation ($\hat{g}_i$).

Table S1: Notations used in the Graph Transformer section. This table summarizes all symbols introduced in the section, along with their descriptions, to aid in understanding the mathematical formulations and model architecture.

Symbol	Description
$G^{(o)}$	Node features for the o-th block of the graph transformer.
$g_i^{(o)}$	Feature vector of the i-th node (gene) in the o-th block.
n	Total number of nodes (genes) in the chromosome.
$q_{h,i}^{(o)}$	Query vector for the h-th attention head at node i in block o.
$k_{h,j}^{(o)}$	Key vector for the h-th attention head at node j in block o.
$v_{h,j}^{(o)}$	Value vector for the h-th attention head at node j in block o.
$W_{h,q}^{(o)}$	Trainable weight matrix for generating query vectors in the h-th attention head of block o.
$b_{h,q}^{(o)}$	Trainable bias vector for generating query vectors in the h-th attention head of block o.
$W_{h,k}^{(o)}$	Trainable weight matrix for generating key vectors in the h-th attention head of block o.
$b_{h,k}^{(o)}$	Trainable bias vector for generating key vectors in the h-th attention head of block o.
$W_{h,v}^{(o)}$	Trainable weight matrix for generating value vectors in the h-th attention head of block o.
$b_{h,v}^{(o)}$	Trainable bias vector for generating value vectors in the h-th attention head of block o.
$e_{ij}^{(o)}$	Edge feature between nodes i and j in block o.
$W_{h,e}^{(o)}$	Trainable weight matrix for encoding edge features in the h-th attention head of block o.
$b_{h,e}^{(o)}$	Trainable bias vector for encoding edge features in the h-th attention head of block o.
$\alpha_{h,ij}^{(o)}$	Final attention score for the edge (i,j) for the h-th attention head in block o.
$\alpha1_{h,ij}^{(o)}$	Edge-only attention score for the h-th attention head in block o.
$\alpha2_{h,ij}^{(o)}$	Node- and edge-combined attention score for the h-th attention head in block o.
$\rho_{h,ij}^{(o)}$	Gated weight combining $\alpha1_{h,ij}^{(o)}$ and $\alpha2_{h,ij}^{(o)}$.
$\langle q, e\rangle$	Exponential dot product between query q and edge e, calculated as $e^{\frac{q^T e}{\sqrt{d}}}$.
$\langle q, k+e\rangle$	Exponential dot product between query q and $k+e$, calculated as $e^{\frac{q^T(k+e)}{\sqrt{d}}}$.
d	Hidden size of each attention head.
$\|\|_{h=1}^{H}$	Concatenation operation across all H attention heads.
$\mathcal{N}(i)$	Neighborhood of node i.
$\hat{g}_i^{(o+1)}$	Aggregated message vector for node i after applying attention.
$r_i^{(o)}$	Residual vector for node i in block o.
$W_r^{(o)}, b_r^{(o)}$	Trainable weight and bias parameters for the residual connection in block o.
$\beta_i^{(o)}$	Gated weight combining residual and attention-aggregated vectors.
$W_g^{(o)}$	Trainable weight matrix for generating $\beta_i^{(o)}$.
$\hat{e}_{ij}^{(o+1)}$	Aggregated edge features for the edge (i,j) in block $o+1$.

2 Results

Table S2: Validation results of hyperparameter search for scHiGex on embryo and brain samples.

Sample	Batch size	Learning rate	Number of heads	ROC AUC
Embryo	8	0.0001	2	0.32197151
	8	0.0001	5	0.841664171
	**8**	**0.0001**	**10**	**0.896012129**
	8	0.0001	11	0.82493844
	8	0.0001	12	0.803052529
	8	0.0001	15	0.845468337
	8	0.0001	20	0.749456869
	1	0.0001	10	0.832456029
	2	0.0001	10	0.854618577
	4	0.0001	10	0.824987287
	16	0.0001	10	0.806799214
	8	0.01	10	0.737529714
	8	0.1	10	0.5
Brain	8	0.1	10	0.59974922
	8	0.01	10	0.829390737
	**8**	**0.0001**	**10**	**0.852770472**
	1	0.0001	10	0.84747924
	2	0.0001	10	0.842675771
	4	0.0001	10	0.830107067
	16	0.0001	10	0.827950948
	8	0.0001	2	0.839259789
	8	0.0001	5	0.833044508
	8	0.0001	11	0.831742693
	8	0.0001	12	0.830233268
	8	0.0001	15	0.822262054
	8	0.0001	20	0.828701855

Table S3: Evaluation results of scHiGex on blind test for embryo cells in ten repeated runs

Model	Accuracy	AP	F1 Score	PCC	MCC	SCC	AUC	Avg. abs. error
1	0.792	0.532	0.474	0.585	0.449	0.405	0.893	0.071
2	0.804	0.543	0.494	0.596	0.468	0.405	0.892	0.068
3	0.806	0.535	0.486	0.585	0.458	0.404	0.891	0.066
4	0.807	0.543	0.499	0.599	0.471	0.404	0.891	0.067
5	0.798	0.525	0.475	0.583	0.449	0.404	0.892	0.067
6	0.803	0.537	0.493	0.590	0.466	0.403	0.890	0.068
7	0.802	0.541	0.492	0.593	0.464	0.404	0.891	0.069
8	0.802	0.531	0.486	0.589	0.489	0.399	0.886	0.068
9	0.801	0.537	0.488	0.587	0.460	0.404	0.891	0.068
10	0.808	0.534	0.494	0.580	0.463	0.404	0.891	0.066

Table S4: Evaluation results of scHiGex on blind test for brain cells in ten repeated runs

Model	Accuracy	AP	F1 Score	PCC	MCC	SCC	AUC	Avg. abs. error
1	0.750	0.538	0.521	0.555	0.436	0.436	0.844	0.097
2	0.750	0.537	0.518	0.555	0.434	0.436	0.844	0.096
3	0.757	0.554	0.530	0.574	0.446	0.439	0.847	0.094
4	0.743	0.571	0.526	0.592	0.444	0.445	0.851	0.098
5	0.763	0.526	0.519	0.536	0.432	0.431	0.840	0.094
6	0.753	0.565	0.531	0.586	0.447	0.441	0.848	0.096
7	0.758	0.571	0.535	0.595	0.451	0.444	0.851	0.094
8	0.768	0.551	0.535	0.568	0.449	0.437	0.845	0.092
9	0.766	0.565	0.539	0.585	0.454	0.442	0.849	0.091
10	0.779	0.505	0.511	0.503	0.418	0.420	0.832	0.094

Table S5: Evaluation results of scHiGex_ind on blind test for embryo cells in ten repeated runs

Model	Accuracy	AP	F1 Score	PCC	MCC	SCC	AUC	Avg. abs. error
1	0.748	0.472	0.419	0.519	0.401	0.496	0.883	0.085
2	0.758	0.477	0.431	0.529	0.409	0.394	0.881	0.083
3	0.762	0.477	0.435	0.529	0.413	0.394	0.881	0.082
4	0.760	0.474	0.443	0.527	0.419	0.389	0.876	0.081
5	0.764	0.484	0.442	0.537	0.419	0.395	0.882	0.080
6	0.762	0.467	0.439	0.526	0.416	0.392	0.879	0.080
7	0.755	0.477	0.423	0.527	0.403	0.395	0.883	0.084
8	0.763	0.488	0.442	0.542	0.421	0.391	0.877	0.081
9	0.762	0.471	0.444	0.527	0.420	0.384	0.871	0.083
10	0.753	0.486	0.418	0.534	0.400	0.399	0.887	0.084

Table S6: Evaluation results of scHiGex_ind on blind test for brain cells in ten repeated runs

Model	Accuracy	AP	F1 Score	PCC	MCC	SCC	AUC	Avg. abs. error
1	0.679	0.514	0.474	0.531	0.392	0.430	0.839	0.122
2	0.684	0.508	0.477	0.522	0.393	0.428	0.838	0.129
3	0.676	0.507	0.470	0.522	0.387	0.428	0.838	0.129
4	0.687	0.513	0.478	0.529	0.395	0.430	0.839	0.123
5	0.694	0.516	0.479	0.532	0.395	0.429	0.839	0.117
6	0.684	0.501	0.472	0.512	0.386	0.421	0.832	0.127
7	0.703	0.513	0.485	0.527	0.399	0.428	0.838	0.115
8	0.693	0.498	0.478	0.507	0.391	0.417	0.829	0.128
9	0.698	0.517	0.485	0.532	0.401	0.430	0.839	0.117
10	0.686	0.505	0.474	0.519	0.389	0.423	0.834	0.123

Table S7: Statistical evaluation results of scHiGex and Naive Predictors.

Sample	Model	Overall Standard Deviation	95% Confidence Interval
Embryo	scHiGex	**0.16**	**(0.084520415, 0.084608264)**
	Naive Predictor 1	0.21	(0.21955499, 0.219672841)
	Naive Predictor 2	0.23	(0.193804507, 0.19393305)
Brain	scHiGex	**0.19**	**(0.109365635, 0.109860532)**
	Naive Predictor 1	0.23	(0.273957929, 0.27454408)
	Naive Predictor 2	0.25	(0.24184554, 0.242494019)

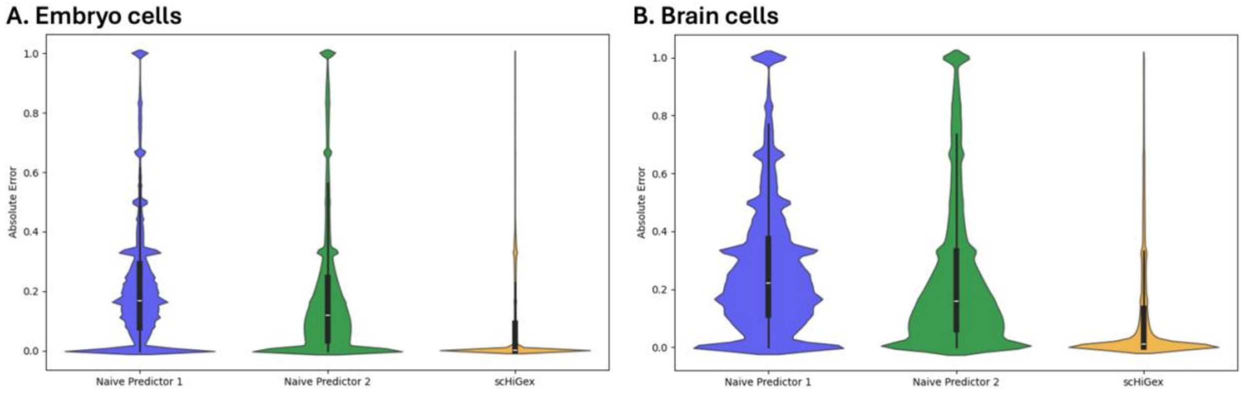

Figure S2: Violin plots showing the distribution of absolute errors for different predictors on two datasets: (A) embryo cells and (B) brain cells.

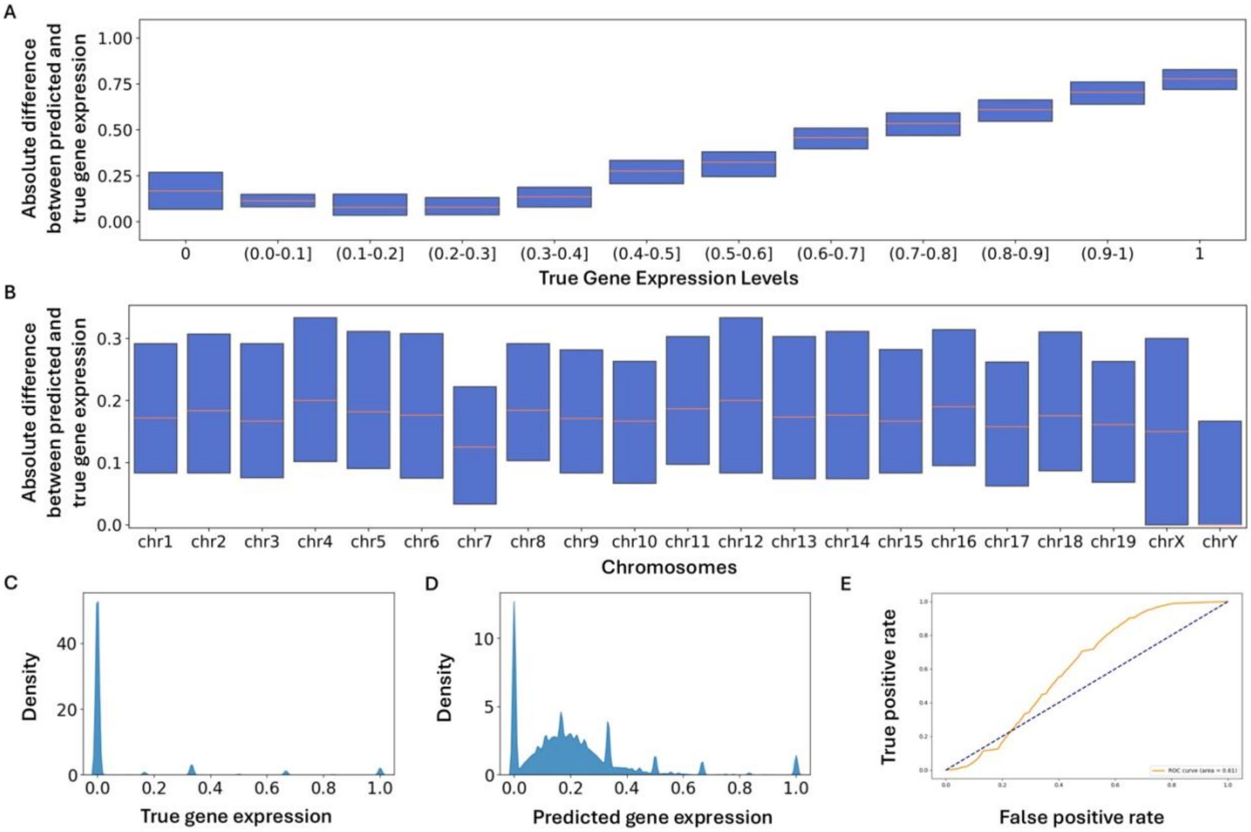

Figure S3: Evaluation plots of blind test on embryo cells using Naïve Predictor 1. (A-B) Box plots of absolute difference between the predicted and true gene expression levels on the blind test cells and each chromosome, respectively. (C-D) Distribution of the true and predicted gene expression levels on the blind test cells. (E) ROC curve of Naïve Predictor 1 on the blind test cells.

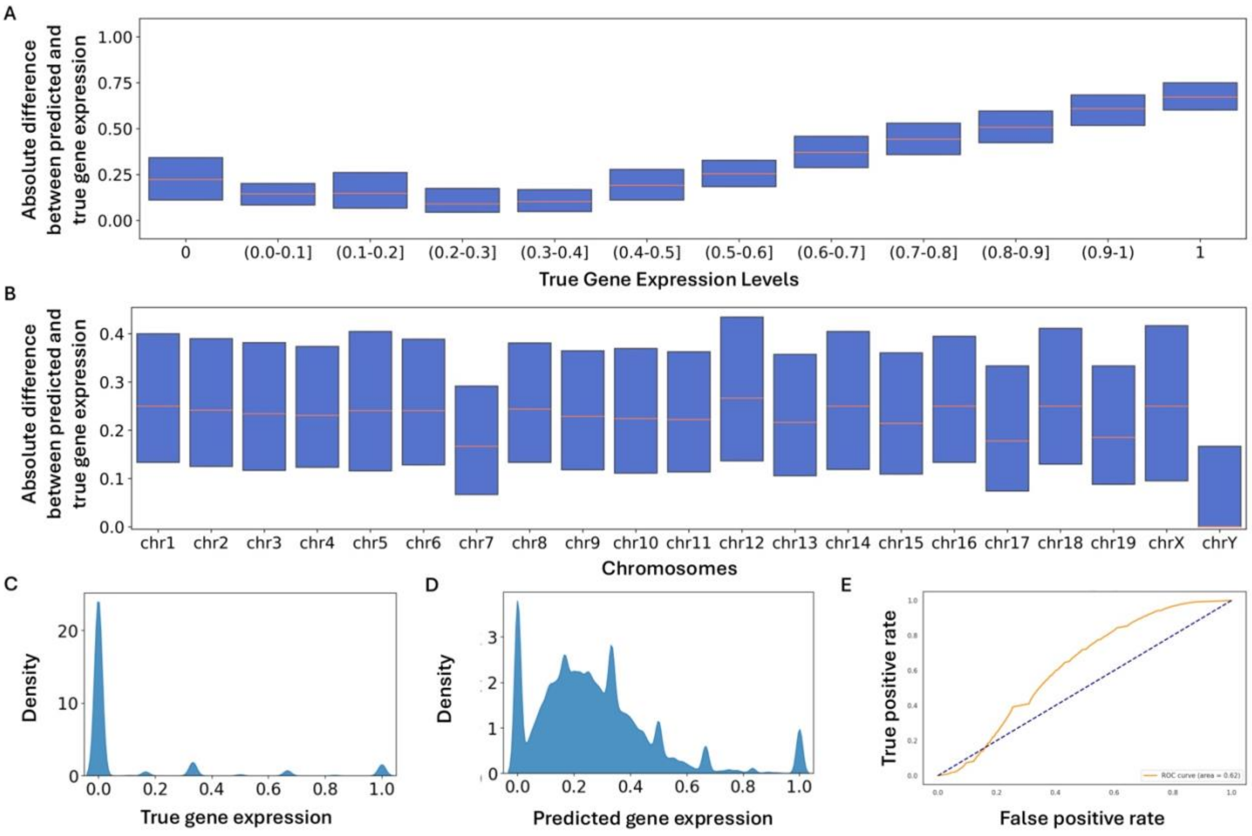

Figure S4: Evaluation plots of blind test on brain cells using Naïve Predictor 1. (A-B) Box plots of absolute difference between the predicted and true gene expression levels on the blind test cells and each chromosome, respectively. (C-D) Distribution of the true and predicted gene expression levels on the blind test cells. (E) ROC curve of Naïve Predictor 1 on the blind test cells.

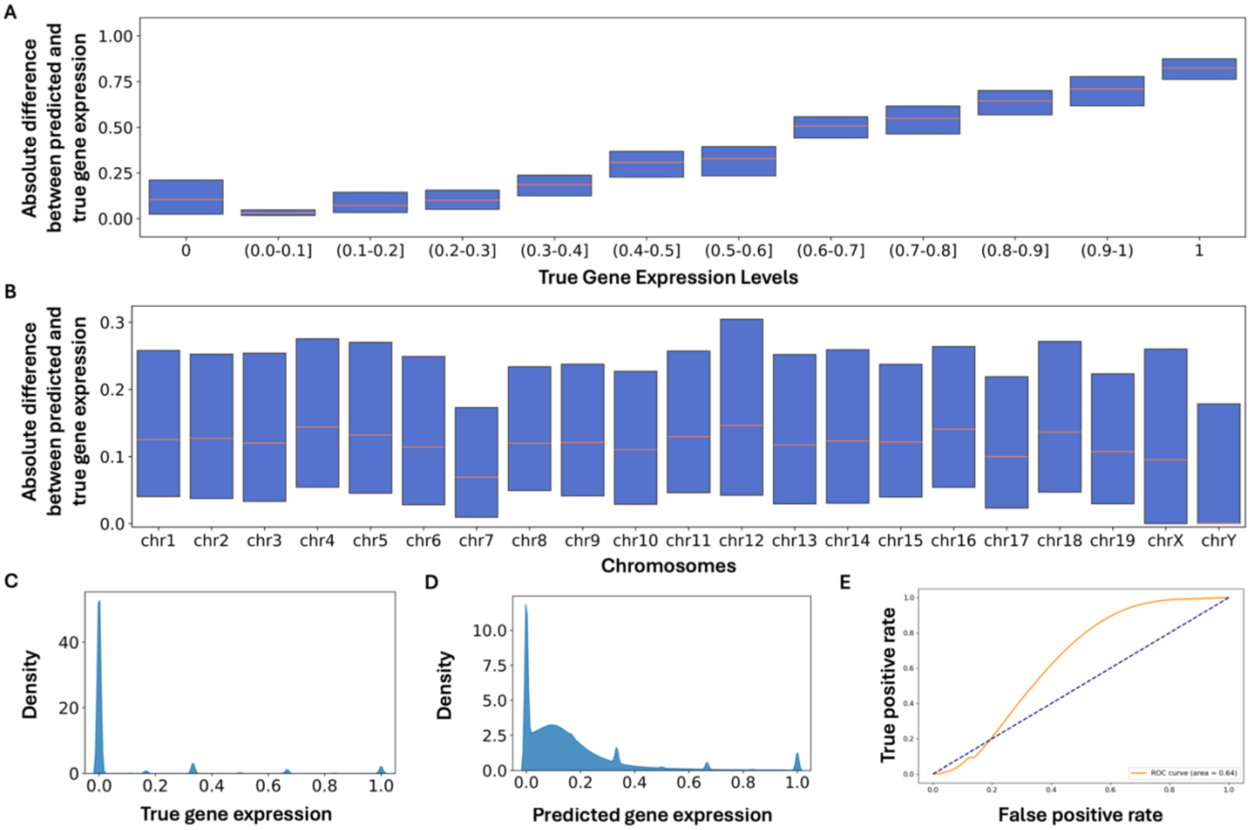

Figure S5: Evaluation plots of blind test on embryo cells using Naïve Predictor 2. (A-B) Box plots of absolute difference between the predicted and true gene expression levels on the blind test cells and each chromosome, respectively. (C-D) Distribution of the true and predicted gene expression levels on the blind test cells. (E) ROC curve of Naïve Predictor 2 on the blind test cells.

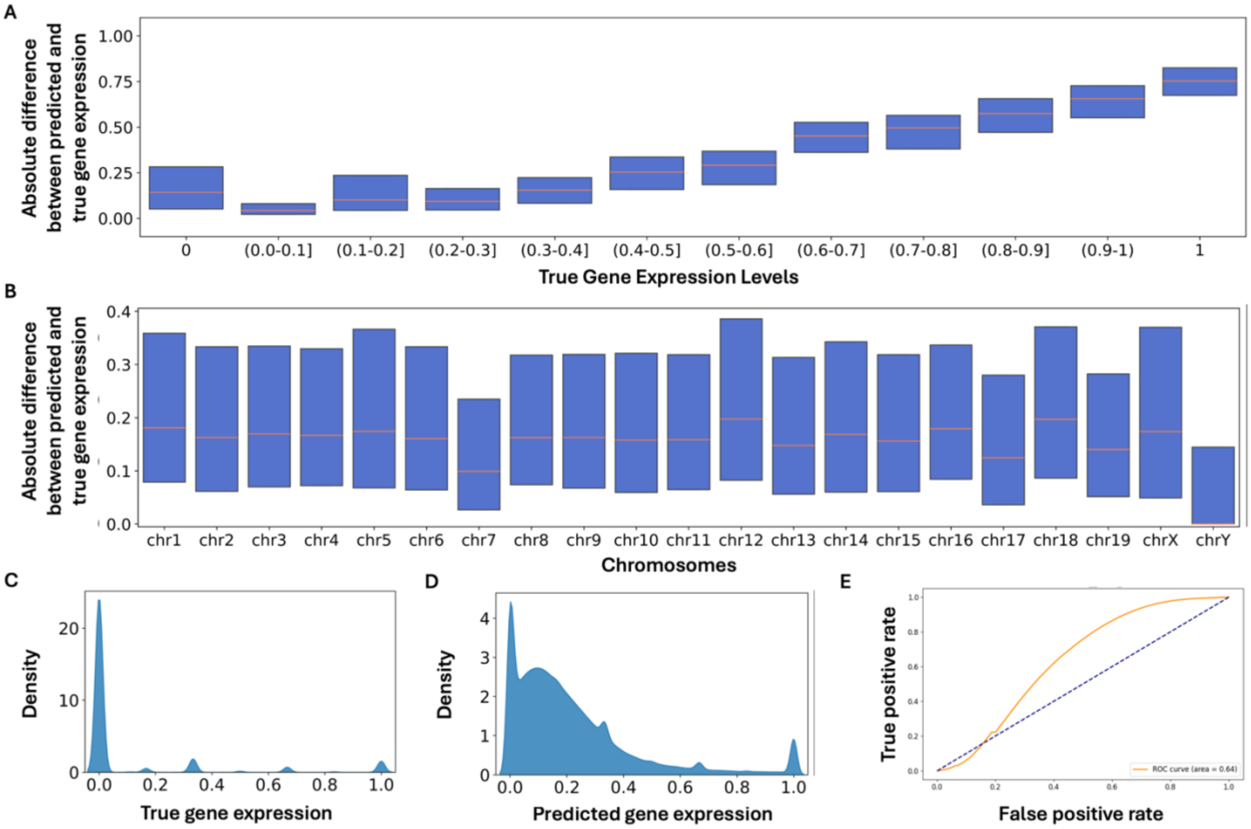

Figure S6: Evaluation plots of blind test on brain cells using Naïve Predictor 2. (A-B) Box plots of absolute difference between the predicted and true gene expression levels on the blind test cells and each chromosome, respectively. (C-D) Distribution of the true and predicted gene expression levels on the blind test cells. (E) ROC curve of Naïve Predictor 2 on the blind test cells.

References

1. Zhou, J., et al., *Robust single-cell Hi-C clustering by convolution-and random-walk–based imputation.* Proceedings of the National Academy of Sciences, 2019. 116(28): p. 14011-14018.
